# Supplementary material for: How Plant Polyhydroxy Flavonoids Can Hinder the Metabolism of Cytochrome 3A4
Source: Biomedicines. 2025 Mar 7;13(3):655. doi: 10.3390/biomedicines13030655 (PMC11940229; doi:10.3390/biomedicines13030655)
Supplement: Supplementary file 1 [file biomedicines-13-00655-s001.zip › biomedicines-3496857-supplementary.pdf]

How Plant Polyhydroxy Flavonoids Can Hinder the Metabolism of Cytochrome 3A4

Carina S. P. Vieira [1], Marisa Freitas [1], Andreia Palmeira [2,3], Eduarda Fernandes [1] and Alberto N. Araújo [1,*]

[1] LAQV, REQUIMTE, Laboratory of Applied Chemistry, Department of Chemical Sciences, Faculty of Pharmacy, University of Porto, 4050-313 Porto, Portugal.

[2] Laboratory of Organic and Pharmaceutical Chemistry (LQOF), Department of Chemical Sciences, Faculty of Pharmacy, University of Porto, Rua de Jorge Viterbo Ferreira, 228, 4050-313 Porto, Portugal

[3] Interdisciplinary Centre of Marine and Environmental Research (CIIMAR), 4450-208 Matosinhos, Portugal

[*] Correspondence: anaraujo@ff.up.pt

Supplementary Materials

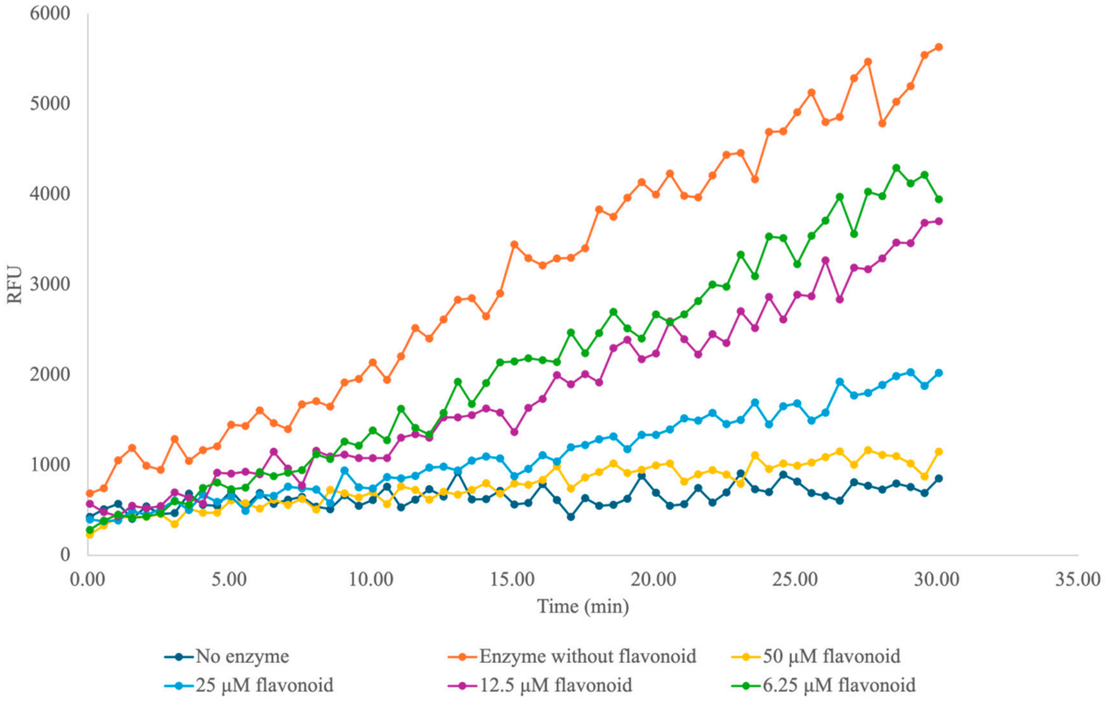

Figure S1. Example of kinetic experiment using the enzyme CYP3A4 and different flavonoid concentrations: 50, 25, 12.5 and 6.25 µM. The blank contained the same constituents as the control, except the enzyme.

Table S1. Effect of the flavonoid substitution pattern on CYP3A4 activity.

**Hydroxylation position or substitution**	**Ring**	**Flavonoid and Effect of the hydroxylation/substituition on the % of inhibition (H → OH)**	**Hydroxylation impact (p value)**
**C5**	A	Resokaempferol (38 ± 7) → Kaempferol (24 ± 8)	0.3527 (ns)
		Fisetin (30 ± 13) → Quercetin (48 ± 9)	0.2327 (ns)
**C6**		Apigenin (23 ± 8) → Scutellarein (76 ± 7)	0.0028 (**)
		Chrysin (28 ± 17) → Baicalein (72 ± 11)	0.0601 (ns)
**C8**		Kaempferol (24 ± 8) → Herbacetin (77 ± 6)	0.0020 (**)
**C2’**	B	Kaempferol (24 ± 8) → Morin (38 ± 11)	0.3374 (ns)
**C3’**		Apigenin (23 ± 8) → Luteolin (69 ± 7)	0.0050 (**)
		Quercetin (48 ± 9) → Myricetin (32 ± 12)	0.2809 (ns)
		Herbacetin (77 ± 6) → Gossypetin (65 ± 7)	0.2762 (ns)
**C4’**		Chrysin (28 ± 17) → Apigenin (23 ± 8)	0.8334 (ns)
		Baicalein (72 ± 11) → Scutellarein (76 ± 7)	0.7662 (ns)
		Galangin (4 ± 9) → Kaempferol (24 ± 8)	0.1504 (ns)
**C5’**		Resokaempferol (38 ± 7) → Fisetin (30 ± 13)	0.6835 (ns)
		Kaempferol (24 ± 8) → Quercetin (48 ± 9)	0.0656 (ns)
		Myricetin (32 ± 12) → Gossypetin (65 ± 7)	0.0282 (*)
**C3**	C	Chrysin (28 ± 17) → Galangin (4 ± 9)	0.2849 (ns)
		Apigenin (23 ± 8) → Kaempferol (24 ± 8)	0.9141 (ns)
		Luteolin (69 ± 7) → Quercetin (48 ± 9)	0.0885 (ns)
**C2 = C3**		Naringenin (-11 ± 5) → Apigenin (23 ± 8)	0.0099 (*)

Each value represents the mean ± SEM of at least three experiments. * $p < 0.05$; ** $p < 0.01$; *** $p < 0.001$; **** $p < 0.0001$.

Table S2. Physico-chemical properties of the studied flavonoids (data obtained from From Swiss Institute of Bioinformatics – Swiss ADME and Chemicalize platform (©Chemaxon).

Flavonoid	MW (g/mol)	Number Hydrogen bond acceptors	Number Hydrogen bond donors	Hydroxyl number	TPSA (Å^2)	pKa	WLOGP	Consensus LogP O/W	Medicinal chemistry
**Flavone**	222.24	2	0	0	30.21	-1.63	3.46	3.18	no alerts
**Chrysin**	254.24	4	2	2	70.67	6.58	2.87	2.55	no alerts
**Apigenin**	270.24	5	3	3	90.9	5.48	2.58	2.11	no alerts
**Baicalein**	270.24	5	3	3	90.9	5.61	2.58	2.24	catechol
**Luteolin**	286.24	6	4	4	111.13	6.57	2.28	1.73	catechol
**Scutellarein**	286.24	6	4	4	111.13	5.61	2.28	1.81	catechol
**Galangin**	302.24	7	5	5	131.36	5.22	1.99	1.23	catechol
**Resokaempferol**	270.24	5	3	3	90.9	5.23	2.58	1.99	no alerts
**Kaempferol**	286.24	6	4	4	111.13	6.29	2.28	1.58	no alerts
**Fisetin**	318.24	8	6	6	151.59	5.22	1.69	0.79	catechol
**Quercetin**	270.24	5	3	3	90.9	5.17	2.58	2.01	no alerts
**Morin**	286.24	6	4	4	111.13	5.17	2.28	1.55	catechol
**Herbacetin**	302.24	7	5	5	131.36	6.2	1.99	1.2	no alerts
**Myricetin**	302.24	7	5	5	131.36	6.79	1.99	1.33	catechol. hydroquinone
**Gossypetin**	318.24	8	6	6	151.59	6.34	1.69	0.96	catechol. hydroquinone
**Naringenin**	272.25	5	3	3	86.99	7.86	2.19	1.84	no alerts

GI: Gastrointestinal; LogP O/W: Octanol-water partition coefficient; pKa: acid dissociation constant; MW: Molecular Weight; TPSA: Topological Polar Surface Area; WLOGP: Water Partition Coeficient.

Suplementary Data S1. Protein–ligand interaction fingerprints (PLIF) of CYP3A4:inhibitor complexes

Protein Ligand Interaction Fingerprints (PLIF) tool [1] implemented in MOE 2022.02 (ChemComp, Canada, Montreal) was used to summarize the intermolecular interactions between inhibitors and CYP3A4 using a fingerprint scheme. 63 Structures of human CYP3A4 bound to diverse inhibitors and with a resolution better than 3 Å were retrieved from Protein Data Bank (PDB codes: 3NXU, 3TJS, 4D6Z, 4D75, 4I4G, 4I4H, 4K9T, 4K9U, 4K9V, 4K9W, 4NY4, 5VC0, 5VCE, 6BCZ, 6BD6, 6BD7, 6BD8, 6BDH, 6BDI, 6BDK, 6BDM, 6DA2, 6DA3, 6DA5, 6DA8, 6DAA, 6DAB, 6DAC, 6DAG, 6DAJ, 6DAL, 6MA8, 6UNE, 6UNG, 6UNH, 6UNI, 6UNL, 6UNM, 7KS8, 7KSA, 7KVH, 7KVI, 7KVJ, 7KVK, 7KVM, 7KVO, 7KVS, 7UAY, 7UAZ, 7UF9, 7UFA, 7UFB, 7UFC, 7UFD, 7UFE, 7UFF, 8EWL, 8EWM, 8EWP, 8EWQ, 8EWS, 8EXB, and 8SPD). The fingerprints for all protein-bound ligands were generated using a minimum energetic cutoff of 0.5 kcal/mol for the analyzed interactions (hydrogen bonds and π interactions). Results were presented in Population Display and Barcode display.

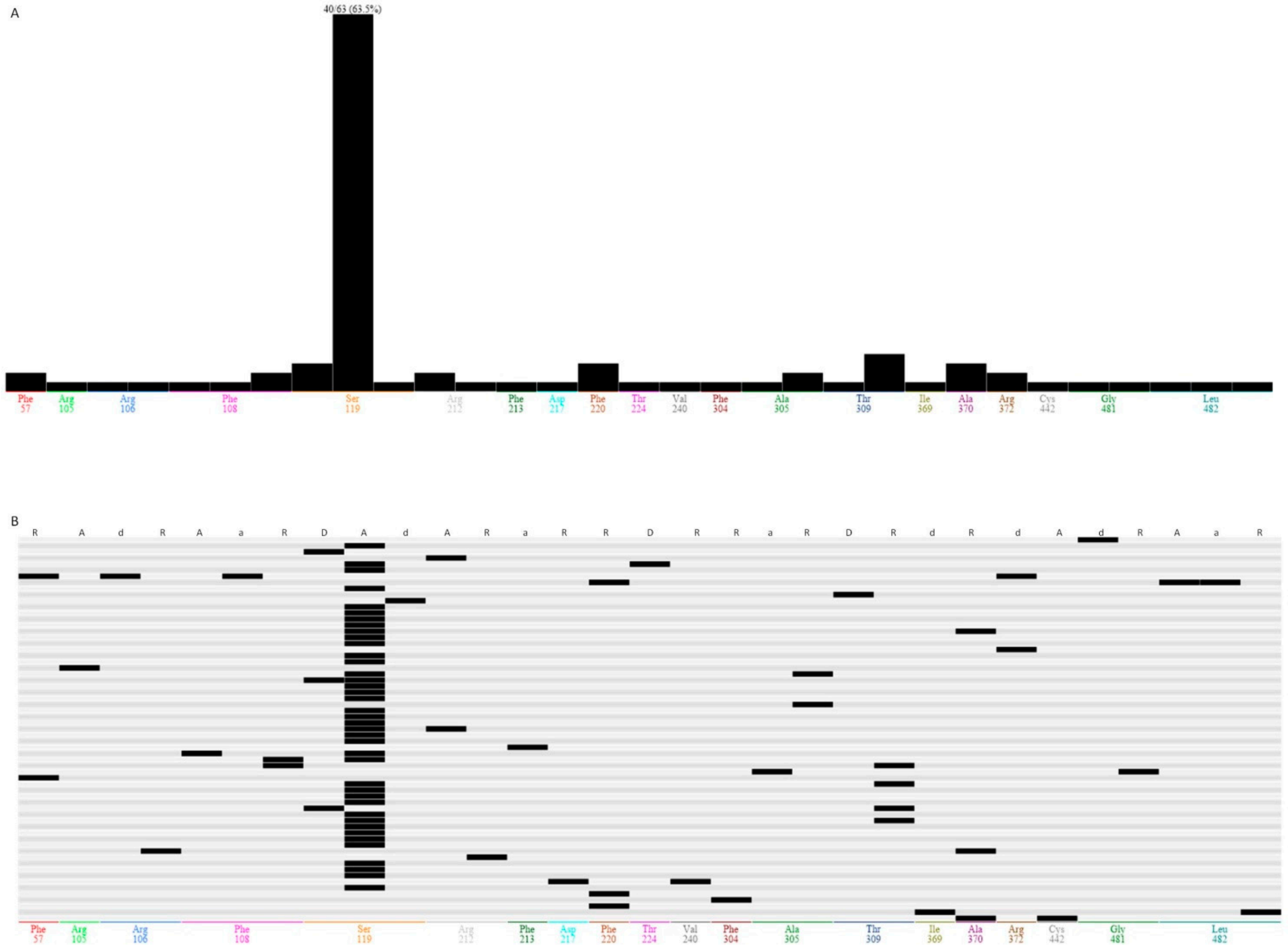

Figure S2. PLIF analysis of CYP3A4-inhibitor complexes. (**A**) Histogram (Population Display) of PLIF results showing the number of ligands (Y-axis) with which each residue (plotted in the X-axis) interacts. Depicted residue Ser-119 has the higher bar (63.5%), meaning that this amino acid establishes interactions with most of the CYP3A4 inhibitors. (**B**) Barcode display analysis of PLIF results. The horizontal rows correspond to each of the studied complexes. A black rectangle indicates the presence of an interaction with residues shown in the X-axis of the graph. A: sidechain hydrogen bond acceptor; a: backbone hydrogen bond acceptor; D: sidechain hydrogen bond donor; d: backbone hydrogen bond donor; R: arene interaction.

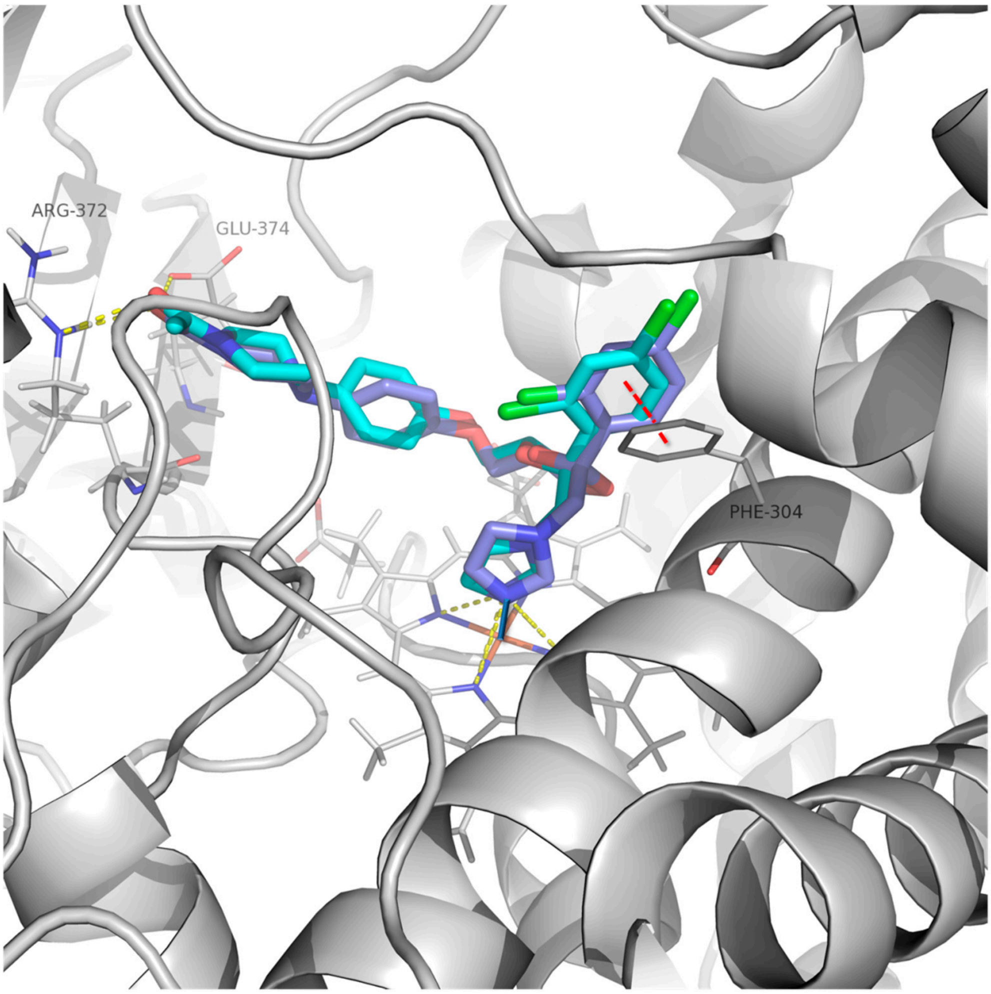

Figure S3. Cristalographic (dark blue sticks) and docked (light blue sticks) ketoconazol. The CYP3A4 target (2V0M) is represented as ribbons. Hydrogen interactions and π- π interactions are represented as yellow and red broken lines. respectively. The residues involved in the bind are represented as thin sticks and labeled. The nitrogen lone pair in the imidazole ring can directly coordinate the heme iron to elicit inhibition (this bond is represented as a blue line) [2,3]. The ketoconazole keto group is located in a polar pocket and hydrogen interactions are established with residues Arg-372 and Glu-374. The interaction is further stabilized by π-stacking between the imidazole ring and the side chain of Phe-304 [4].

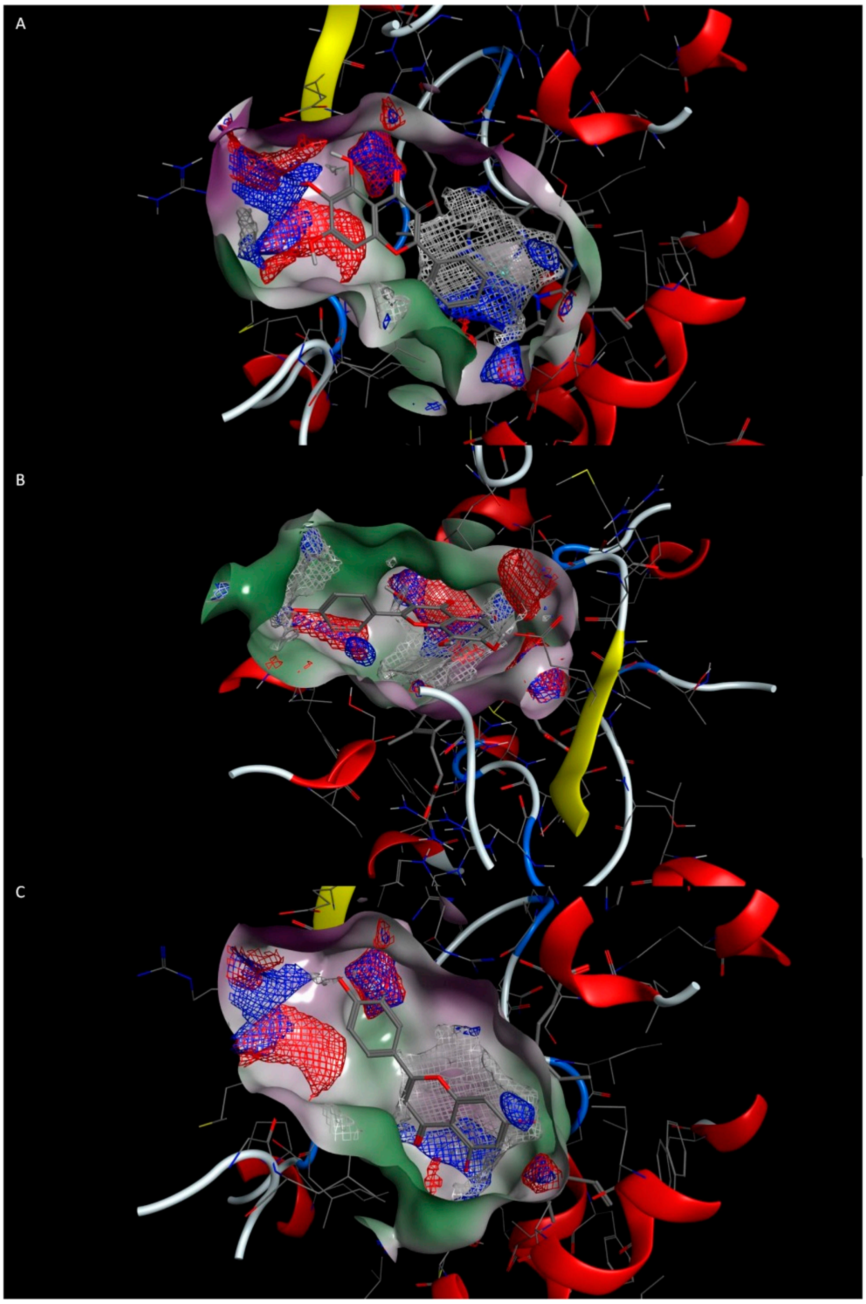

Figure S4. Interaction potential maps of (A) baicalein, (B) herbacetin and (C) luteolin docked into CYP3A4 binding site. The interaction potential maps are represented as a net for better visualization. The interaction potential map provides a graphical representation of where a chemical probe has favorable interactions with a molecular surface, based on a force field incorporating van der Waals, charge and hydrogen bonding terms. The interaction potential surface comprises three maps associated with three probe types. The interaction potentials are displayed for the values of -5.5 kcal/mol for the OH2 probe,

which represents the hydrophilic regions (red net), and -2.5 kcal/mol for the N probe, which represents the hydrogen bond acceptors (blue net), and DRY probe, which represents the hydrophobic regions (grey net).

By examining the interaction potential map, it is noticed that the aromatic portions of the ligand mainly occur in the hydrophobic regions of the interaction potential map, whereas the heteroatoms of the ligand occur mainly on the hydrophilic regions of that map. By comparing these results to that obtained by coloring a molecular surface by lipophilicity (solid surface with hydrophilic region coloured in purple, neutral region coloured in white, and lipophilic region coloured in green color), it is noticeable that the OH2 probe surfaces (red net) tend to occur near the hydrophilic regions (purple solid surface).

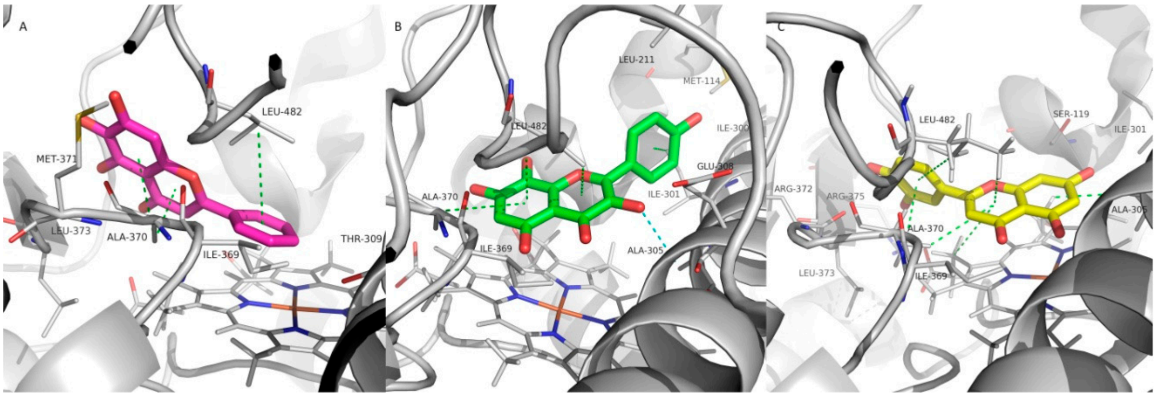

Figure S5. Docking of flavones into CYP3A4 – other contacts. Detailed view of top ranked poses of (A) baicalein (pink sticks), (B) herbacetin (green sticks), and (C) luteolin (yellow sticks) on the active site of CYP3A4. π-Alkyl interactions and C-H bonds are represented as green and blue broken lines, respectively, and the involved residues are represented as thin sticks and labeled. Additional residues represented as thin sticks and labeled are involved in van der Waals interactions.

Table S3. Molecular docking score of top ranked test compounds, residues involved in diferente types of interactions and respective bond lengths.

<table>
<tr><th>Compound</th><th>Docking Score (Kcal.mol^{-1})</th><th>H-bonds (distance in Å)</th><th>Cation-π interaction (distance in Å)</th><th>π-π interactions (distance in Å)</th><th>π -alkyl interactions (distance in Å)</th><th>C-H bond (distance in Å)</th><th>van der Waals interactions</th></tr>
<tr><td rowspan="4">Baicalein</td><td rowspan="4">-8.4</td><td>Arg-372 (3.72)</td><td rowspan="4">Heme (3.93)</td><td rowspan="4">Heme (3.83, 5.06, 4.89)</td><td rowspan="2">Leu-482 (4.13)</td><td rowspan="4"></td><td>Thr-309</td></tr>
<tr><td>Glu-374 (3.30)</td><td>Ile-369</td></tr>
<tr><td>Gly-481 (3.05)</td><td rowspan="2">Ala-370 (3.86)</td><td>Met-371</td></tr>
<tr><td>Arg-105 (3.07)</td><td>Leu-373</td></tr>
<tr><td rowspan="6">Herbacetin</td><td rowspan="6">-8</td><td rowspan="6">Thr-309 (3.20)</td><td rowspan="6"></td><td rowspan="2">Phe-304 (3.61)</td><td>Ile-301 (4.37)</td><td rowspan="6">Ala-305 (2.55)</td><td>Met-114</td></tr>
<tr><td>Ala-370 (4.59)</td><td>Leu-200</td></tr>
<tr><td rowspan="4">Heme (4.29)</td><td rowspan="4">Leu-482 (4.78, 5.09)</td><td>Leu-211</td></tr>
<tr><td>Ile-300</td></tr>
<tr><td>Glu-308</td></tr>
<tr><td>Ile-369</td></tr>
<tr><td rowspan="6">Luteoline</td><td rowspan="6">-8.8</td><td rowspan="2">Arg-105 (3.01)</td><td rowspan="6">Heme (3.92)</td><td rowspan="6">Heme (3.85, 3.89, 5.12, 4.95)</td><td>Ala-305 (4.89)</td><td rowspan="6"></td><td>Ser-119</td></tr>
<tr><td>Ala-370 (4.16)</td><td>Ile-301</td></tr>
<tr><td rowspan="4">Arg-372 (2.98)</td><td rowspan="2">Leu-482 (5.25, 5.45)</td><td>Arg-372</td></tr>
<tr><td>Leu-373</td></tr>
<tr><td rowspan="2">Heme (5.34)</td><td>Ile-369</td></tr>
<tr><td>Arg-375</td></tr>
</table>

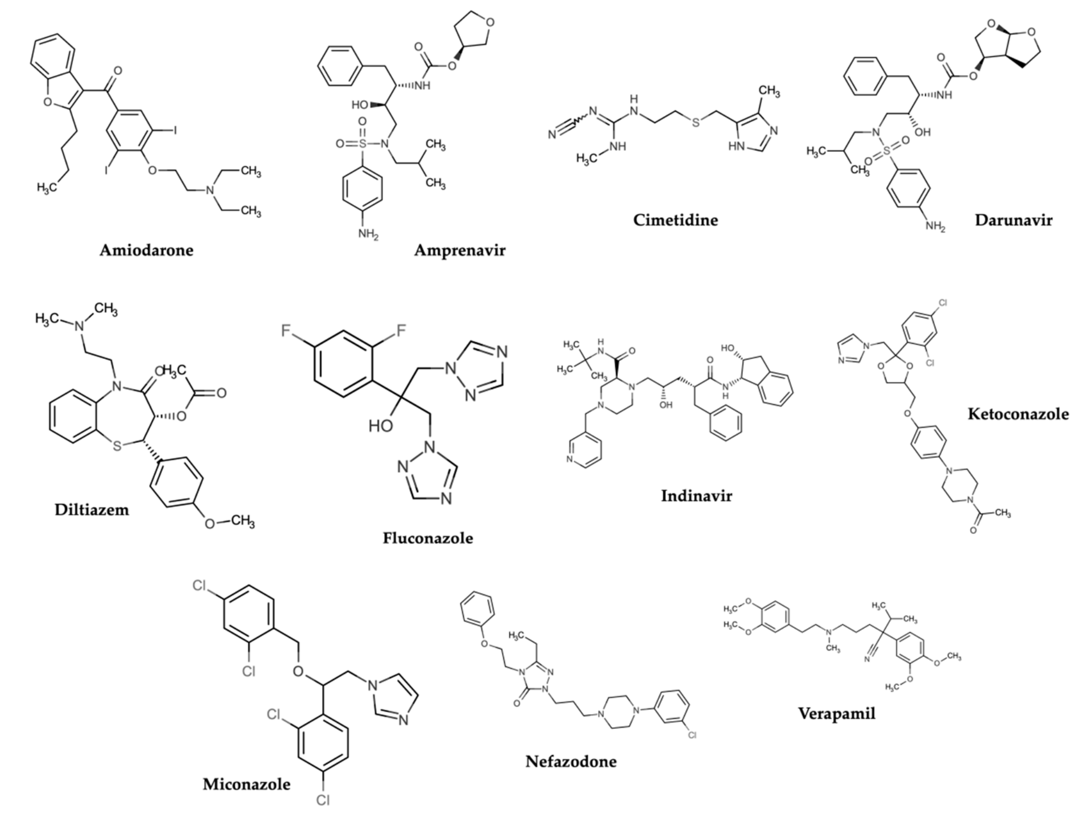

Figure S6. Inhibitor structures used as positive controls in docking studies.

References

1. Da, C.; Kireev, D. Structural Protein-Ligand Interaction Fingerprints (SPLIF) for Structure-Based Virtual Screening: Method and Benchmark Study. *J. Chem Inf. Model.* **2014**, *54*, 2555-2561. https://doi.org/10.1021/ci500319f.

2. Basheer, L.; Kerem, Z. Interactions between CYP3A4 and Dietary Polyphenols. *Oxidative Med. Cell. Longev.* **2015**, *2015*, 854015. https://doi.org/10.1155/2015/854015.

3. Godamudunage, M.P.; Grech, A.M.; Scott, E.E. Comparison of Antifungal Azole Interactions with Adult Cytochrome P450 3A4 versus Neonatal Cytochrome P450 3A7. *Drug Metab. Dispos.* **2018**, *46*, 1329-1337. https://doi.org/10.1124/dmd.118.082032.

4. Ekroos, M.; Sjögren, T. Structural basis for ligand promiscuity in cytochrome P450 3A4. *Proc. Natl. Acad. Sci. U. S. A.* **2006**, *103*, 13682-13687. https://doi.org/10.1073/pnas.0603236103.
